# 1,25-Dihydroxyvitamin D3 alleviates hyperandrogen-induced ferroptosis in KGN cells

**DOI:** 10.1007/s42000-023-00439-5

**Published:** 2023-03-08

**Authors:** Yijie Jiang, Jianshu Yang, Ke Du, Kaiming Luo, Xin Yuan, Fei Hua

**Affiliations:** 1 Department of Endocrinology and Metabolism, The First People’s Hospital of Changzhou, The Third Affiliated Hospital of Soochow University, Changzhou, 213003 China; 2 Health Management Center, The First Affiliated Hospital of Soochow University, Suzhou, 215000 China

**Keywords:** 1,25-dihydroxyvitamin D3, Dehydroepiandrosterone, Ferroptosis, Ovarian granulosa cells

## Abstract

**Purpose:**

Hyperandrogenism, one of the most frequent causes of anovulation in women, increases the risk of metabolic disorders in patients with polycystic ovary syndrome (PCOS). Ferroptosis, characterized by iron-dependent lipid peroxidation, has provided new insight into the progression of PCOS. 1,25-dihydroxyvitamin D3 (1,25D3) may play a role in reproduction because its receptor, VDR, which contributes to the inhibition of oxidative stress, is primarily located in the nuclei of granulosa cells. This study has therefore investigated whether 1,25D3 and hyperandrogenism affect granulosa-like tumor cells (KGN cells) through ferroptosis.

**Methods:**

KGN cells were treated with dehydroepiandrosterone (DHEA) or pretreated with 1,25D3. Cell viability was evaluated with the cell counting kit-8 (CCK-8) assay. The mRNA and protein expression levels of ferroptosis-related molecules, including glutathione peroxidase 4 (GPX4), solute carrier family 7 member (SLC7A11), and long-chain acyl-CoA synthetase 4 (ACSL4), were assessed via qRT–PCR and western blot. The concentration of malondialdehyde (MDA) was measured by ELISA. The rates of reactive oxygen species (ROS) production and lipid peroxidation were assessed via photometric methods.

**Results:**

Decreased cell viability, suppression of GPX4 and SLC7A11 expression, increased expression of ACSL4, elevated levels of MDA, accumulation of ROS, and increased lipid peroxidation, which are changes representative of ferroptosis, were observed in KGN cells after treatment with DHEA. Pretreatment with 1,25D3 in KGN cells significantly prevented these changes.

**Conclusions:**

Our findings demonstrate that 1,25D3 attenuates hyperandrogen-induced ferroptosis of KGN cells. This finding might lead to new insights into the pathophysiology and therapy of PCOS and provides new evidence for the treatment of PCOS with 1,25D3.

## Introduction

Polycystic ovary syndrome (PCOS) is the most common endocrinopathy in women of reproductive age, with a prevalence from 4 to 21% [3], and is accompanied by hyperandrogenism, ovulatory dysfunction, insulin resistance, obesity, and abnormal lipid metabolism [9]. Most women (60–80%) with PCOS present with hyperandrogenism and excessive levels of circulating androgens [13]. Long-term and ongoing exposure to hyperandrogen may disrupt follicular development. The etiology of PCOS is still unknown; therefore, it is essential to clarify its pathogenesis and thereby develop therapeutic interventions.

Ferroptosis is a newly discovered regulated cell death caused by iron-dependent lipid peroxidation; it is genetically, biochemically, and morphologically different from other forms of regulatory cell death, such as apoptosis, necroptosis, and pyroptosis [17]. During ferroptosis, the disrupted function of the cystine/glutamate inverse transporter (System xc-) decreases glutathione production (GSH), preventing GPX4 from performing its normal antioxidant function [20]. Therefore, cell lipid membranes, which are enriched with phospholipids, are extremely susceptible to ROS attack. The end products of lipid peroxidation, including MDA, easily form adducts with proteins and DNA, causing substantial cytotoxic effects and inducing cellular ferroptosis [20]. System xc-, GPX4, SLC7A11, and ACSL4 are key players in ferroptosis. Ferroptosis has been reported in multiple diseases, such as cancer, stroke, ischemic heart disease, and organ transplantation [5]. However, ferroptosis has been less extensively studied in PCOS.

1,25-dihydroxyvitamin D3 (1,25D3), an active form of vitamin D (VD), also known as calcitriol, is a fat-soluble secosteroid hormone that plays a pleiotropic role in a wide range of biological functions [16]. It plays a major role in calcium homeostasis, bone metabolism, and cell differentiation, proliferation, and apoptosis. Among the many physiological processes influenced by VD, a vital role for it in reproduction physiology has been proposed [12]. VD supplementation has beneficial effects on menstrual dysfunction in women suffering from PCOS [18]. However, little is known about the mechanisms by which VD deficiency affects PCOS. VD has been shown to regulate iron metabolism and modulate the hepcidin–ferroportin axis in both humans and other mammals [1]. Deficiency in 1,25D3 is a cause of mitochondrial dysfunction, and an increase in the level of 1,25D3 has been associated with increased mitochondrial performance, such as oxidative phosphorylation [7, 14]. Lower expression and activity of GPx and lower ROS production were observed in polycystic ovary granulosa cells but not normal human granulosa cells, and these effects were attenuated by means of VD treatment [11]. Ferroptosis may be involved in the action of 1,25D3 on ovarian granulosa cells.

Androgen excess is a key pathogenetic mechanism in PCOS. Ovarian granulosa cells (GCs) are critical in folliculogenesis. A previous study showed that maternal hyperandrogenism and insulin resistance induced ferroptosis in the gravid uterus and placenta [21], suggesting a potential role for androgen in the activation of ferroptosis. However, it is not known whether androgen alone can induce ferroptosis in granulosa cells and whether 1,25D3 interferes with this process. Therefore, we aimed to investigate whether androgen is involved in the regulation of ferroptosis in granulosa cells and to determine whether supplemental 1,25D3 confers a protective effect against DHEA-associated ferroptosis of KGN cells.

## Methods

### Cell culture and treatment

The KGN human granulosa-like tumor cell line (FH1125, Fuheng Biology, Shanghai, China) exhibits the physiological characteristics of ovarian cells. The cells were grown in DMEM/F12 (BasalMedia, L310KJ, Shanghai, China) supplemented with 10% fetal bovine serum (FBS, BI, 04-001-1ACS, Israel) and 1% penicillin/streptomycin (Gibco, 15140-122, Thermo Fisher Scientific, USA). All the cells were cultured in humidified atmosphere containing 5% CO_2_ at 37 °C. Twenty-four hours after seeding, the VD cell group was pretreated with 100 nM 1,25-dihydroxyvitamin D3 (MCE, HY-10002) dissolved in absolute dimethyl sulfoxide (DMSO, 0.1%) for 24 h. The medium was then removed, and the cells were treated with 10 μM dehydroepiandrosterone (DHEA, Sigma) dissolved in absolute DMSO (0.1%) for 24 h. The D group was treated with the same concentration of DMSO for 24 h and then with DHEA for 24 h. The control group were treated with the same concentration of DMSO (0.1%) each time the medium was changed.

### CCK-8

Cell viability was measured using cell counting kit 8 (CCK-8, Beyotime, China) at 24 h and 48 h, according to the manufacturer’s instructions. Cells were seeded in 96-well plates at a density of 3000 cells per well and exposed to various concentrations of the indicated compounds for the noted times. Ten microliters of working reagent was added to each well and incubated for 3 h at 37 °C. The absorbance was measured at a wavelength of 450 nm with a BioTek Elx 800 (BioTek, USA).

### RNA extraction and qRT–PCR

Total RNA was extracted from cells using TRIzol Reagent (Invitrogen, USA) according to the manufacturer’s specifications. The purity and concentration of RNA were spectrophotometrically analyzed using a Nanodrop One (Thermo Fisher, USA). To determine the expression of mRNAs, cDNA was transcribed using a PrimeScript RT reagent kit (Takara, Japan). We performed quantitative real-time PCR (qRT–PCR) to quantify the mRNA levels of GPX4, SLC7A11, and ACSL4 with SYBR Green PCR Master Mix (Takara, Japan) on an ABI 7500 Real-Time PCR system (Thermo Fisher, USA). GAPDH was the endogenous control for mRNA. The relative expression was calculated using the 2^−△△CT^ method; the primers are listed in Table 1.

**Table 1:** Primer sequences for qRT-PCR

	Primer sequences
GAPDH	F: 5′CGCATCTTCTTTTGCGTCG3′
	R: 5′TTGAGGTCAATGAAGGGGTCA3′
GPX4	F: 5′GAGGCAAGACCGAAGTAAACTAC3′
	R: 5′CCGAACTGGTTACACGGGAA3′
SLC7A11	F: 5′TGCTGGGCTGATTTTATCTTCG3′
	R: 5′GAAAGGGCAACCATGAAGAGG3′
ACSL4	F: 5′TCTGCTTCTGCTGCCCAATT3′
	R: 5′CGCCTTCTTGCCAGTCTTTT3′

### Western blot

The cells were washed once with ice-cold PBS and lysed with RIPA lysis buffer (Beyotime, China). Total protein was extracted from the cells in the presence of protease inhibitors (Thermo Fisher, USA), and the protein concentration was quantified using a BCA protein assay kit (Beyotime, China). SDS–PAGE was performed to separate the proteins (14.4 μg protein per lane). The gel was then transferred onto a polyvinylidene difluoride membrane (Thermo Fisher, USA). After blocking in 0.1% tris-buffered saline tween (TBST) that contained 5% skim milk for 1 h at room temperature, the PVDF membrane was incubated overnight at 4 °C with primary antibodies against GPX4 (52455S; CST, 1:1000), SLC7A11 (12691S; CST, 1:1000), ACSL4 (22401-1-AP; Proteintech, 1:1000), and β-actin (4970; CST, 1:1000). The membrane was rinsed three times with TBST and then incubated with a specific secondary antibody (7074; CST, 1:3000) BPA-disturbed for 1 h at room temperature. The membrane was washed, and the protein levels were detected with an ECL detection solution (Thermo Fisher Scientific, Waltham, Massachusetts, USA), visualized on a ChemiDoc Touch Imaging System (Bio–Rad, USA), and quantified with the Image J software v1.49.

### Determination of oxidative stress and iron contents

The MDA concentrations were determined with an MDA assay kit (Beyotime, China). A sufficient amount of working reagent was prepared according to the manufacturer’s instructions. After reagent treatment of cells in a 96-well plate, the cell supernatants were collected and their optical density measured at 532 nm, which was used for the calculation of the MDA concentration. SOD, NO, GSH, hydroxyl radical, and Fe2+ contents were determined by commercially available kits provided by Beyotime.

### ROS assay

Intracellular ROS levels were evaluated with 2′,7′-dichlorofluorescein-diacetate (Beyotime, China) according to the manufacturer's guidelines. In summary, KGN cells from each group were cultured in 48-well plates and then incubated with 500 μL of 75 μM H2DCF-DA for 20 min in the dark at 37 °C. The incubated cells were subsequently washed three times with serum-free medium. Hoechst (Beyotime, China) stain was generated as a working solution and added to cells that had been treated as indicated. After incubation at 37 °C for 5 min, the cells were washed three times with serum-free medium, and images were taken with an IX71 microscope (Olympus, USA) in three randomly selected fields.

### Evaluation of the lipid peroxidation rate

The experiments were performed with cells in 48-well plates. To avoid edge effects, the outermost rows of wells were not used. KGN cells were seeded at a density of 5000 cells per well. The cells were preincubated, as described below, with probes before oxidants were added. C11-BODIPY581/591 (Invitrogen™ D3861) was dissolved in DMSO (1 mg in 200 μL of DMSO) and diluted in the medium to the required concentration (5 μM). KGN cells were incubated in 500 μL of 5 μM BODIPY581/591 C11 in the dark in a humidified atmosphere with 5% CO_2_ in the air for 30 min. Then, the cells were washed three times in serum-free medium. Hoechst (Beyotime, China) stain was formulated as a working solution and added to cells that had been treated as indicated. After incubation at 37 °C for 5 min, the cells were washed three times with serum-free medium, and images were taken with an IX71 microscope (Olympus, USA). The photographs were analyzed, as described above.

### Statistical analysis

All statistical analyses were performed using GraphPad Prism software, and the significance of the means and standard error or standard deviation were analyzed with Student’s *t* test for pairwise comparisons or ANOVA for multivariate analyses. A *P* value < 0.05 was considered to be statistically significant (*P < 0.05; **P < 0.01; ***P < 0.001).

## Results

### Effect of different concentrations of DHEA and 1,25-dihydroxyvitamin D3 on the viability of KGN cells

**Fig. 1:**
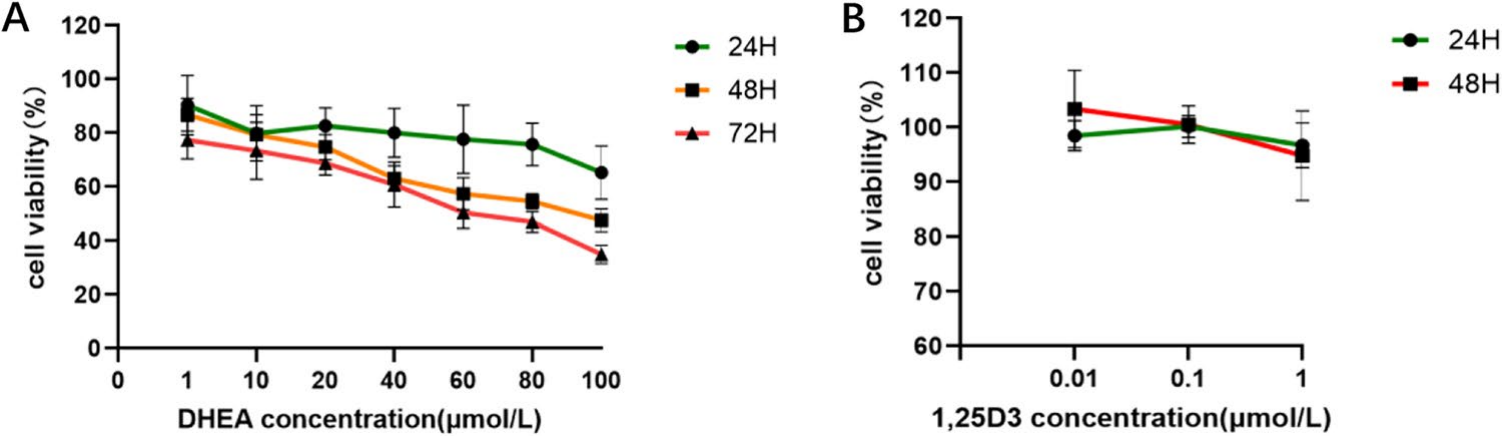
The viability of KGN cells being cultured in the media with various DHEA concentrations for 24, 48, and 72 h, respectively (**a**). The viability of KGN cells being cultured in the medium with different concentrations of 1,25D3 for 24 and 48 h (**b**)

Cell viability was measured in medium with various DHEA concentrations (1, 10, 20, 40, 60, 80, and 100 μmol/L) at 24 h, 48 h, and 72 h. Compared with that of KGN cells treated with 1 μmol/L DHEA, the viability of cells cultured in medium with 10 μmol/L DHEA was lower. However, cell viability was decreased as the DHEA concentration further increased (Fig. 1a). After being treated with the different concentrations of 1,25D3 at 24 h and 48 h, cell viability was increased in the 0.1 μmol/L 1,25D3-treated groups (Fig. 1b).

### 1,25-dihydroxyvitamin D3 attenuated the reduction in cell viability induced by HDEA treatment

**Fig. 2:**
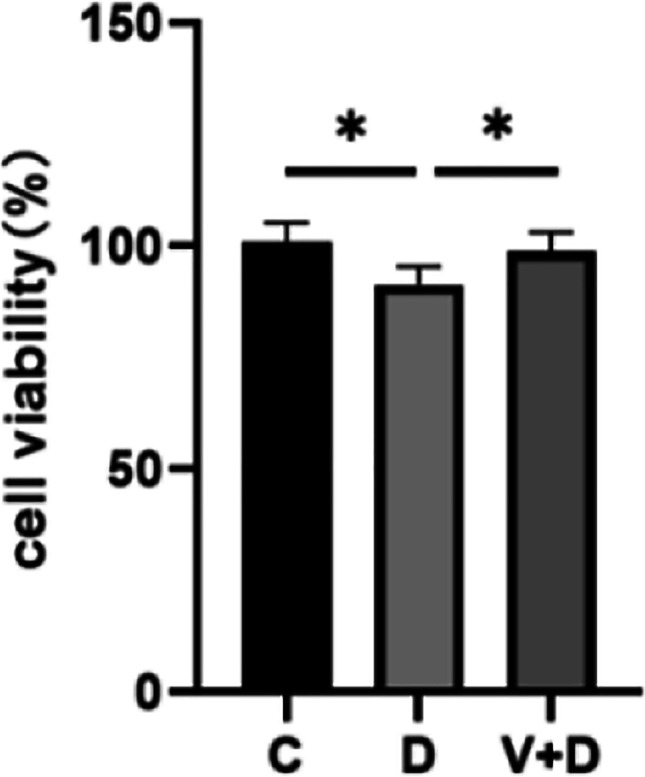
1,25D3 alleviates reduced cell viability in KGN cells after DHEA treatment. Data are shown as mean ± SD. *P < 0.05

As illustrated in Fig. 2, CCK-8 assays showed that DHEA inhibited KGN cell proliferation, whereas pretreatment with 1,25-dihydroxyvitamin D3 increased KGN cell viability.

### 1,25-dihydroxyvitamin D3 ameliorated DHEA-induced alterations in ferroptosis-related gene expression

**Fig. 3:**
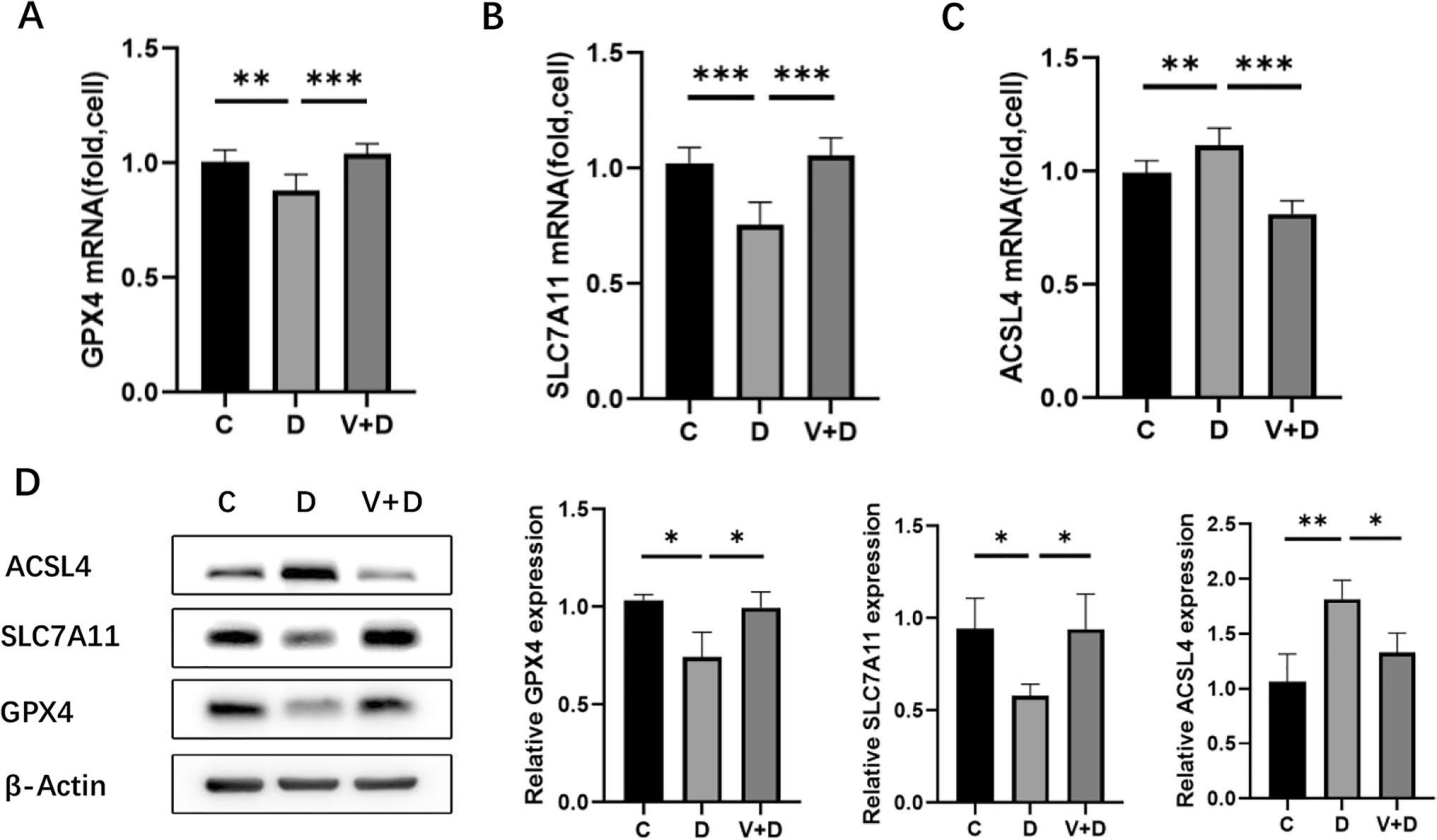
1,25D3 alleviated DHEA-induced ferroptosis-related gene alterations in KGN cells. The mRNA expression levels for GPX4 (**a**), SLC7A11 (**b**), and ACSL4 (**c**) in each group of cells. **d** Western blot analysis of GPX4, SLC7A11, and ACSL4. The results are representative of three independent experiments. Data are presented as mean ± SD, *P < 0.05 , ** *P* < 0.01, ***P < 0.001

The mRNA expression of GPX4 (Fig. 3a) and SLC7A11 (Fig. 3b) were decreased, and the expression of ACSL4 (Fig. 3c) was increased in D cells compared with C cells. In addition, the western blot results showed a change in the protein expression levels of GPX4, SLC7A11, and ACSL4 that paralleled the changes in the corresponding mRNAs (Fig. 3d). Decreased expression of GPX4 (Fig. 3a and d) and SLC7A11 (Fig. 3b and d), as well as increased expression of ACSL4 (Fig. 3c and d), caused by ferroptosis, were significantly reversed after treatment with 1,25D3.

### 1,25-dihydroxyvitamin D3 regulates ferroptosis-induced oxidative stress after exposure to high androgen levels

**Fig. 4:**
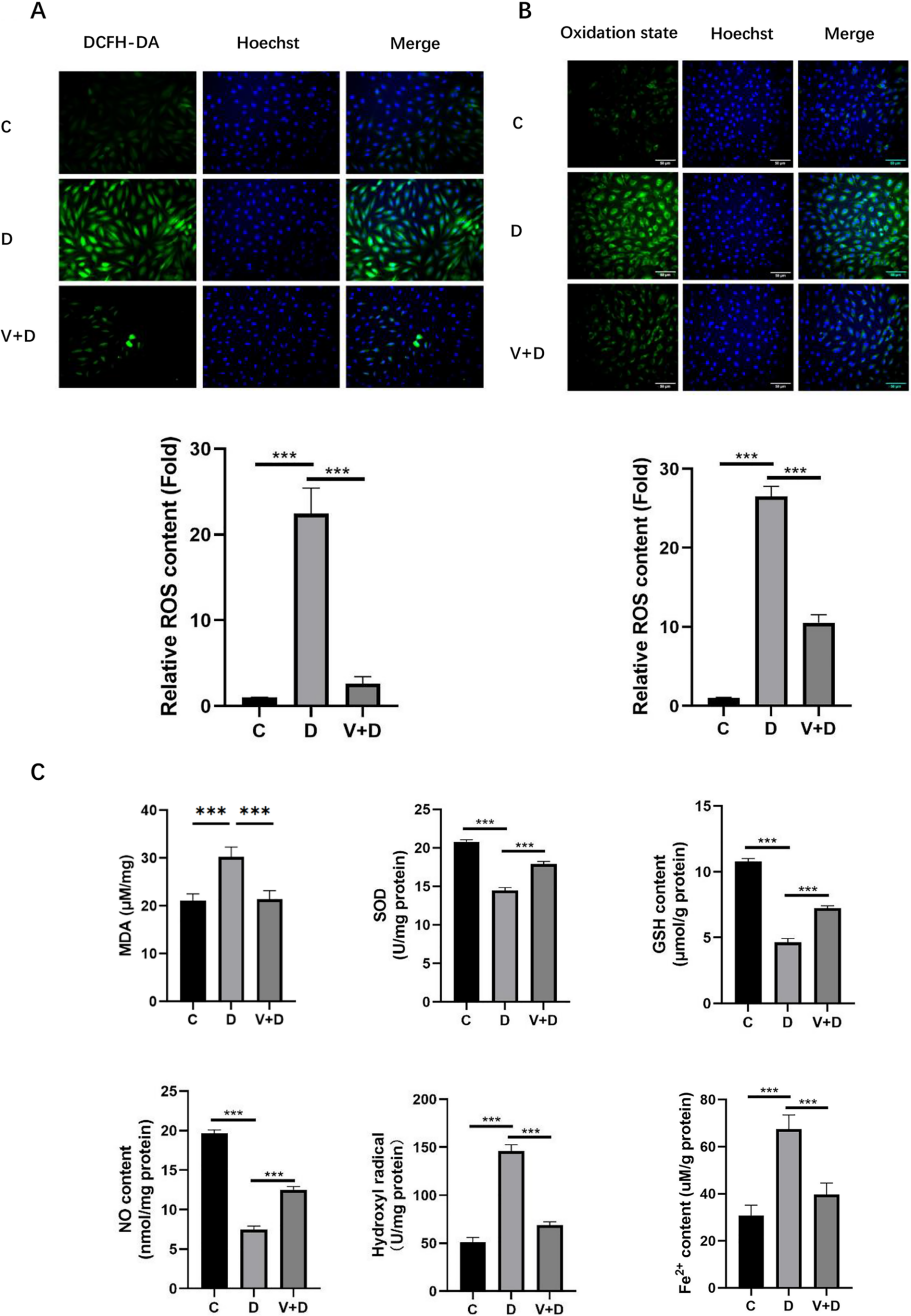
1,25D3 inhibits oxidative stress induced by DHEA in KGN cells. **a** The production of ROS (green), nuclear staining with Hoechst (blue) in each group of cells. **b** Lipid peroxidation, oxidation state (green) visualized by 5 μM C11-BODIPY581/591 staining in KGN cells, and nuclear staining with Hoechst (blue) in each group of cells. **c** The malondialdehyde (MDA), superoxide dismutase (SOD), nitric oxide (NO), glutathione (GSH), hydroxyl radicals, and Fe^2+^ content in KGN cells. Data are shown as mean ± SD. *P < 0.05, **P < 0.01, ***P < 0.001

Ferroptosis is characterized by excessive oxidative stress leading to lipid peroxidation. KGN cells induced by DHEA were stained with 2′,7′-dichlorofluorescein diacetate to determine the rate of ROS generation. The accumulation of ROS triggered by DHEA emitted stronger fluorescence compared to that emitted by the C group, and the high intensity of fluorescence was effectively reduced by pretreatment with 1,25D3 (Fig. 4a). There was minimal background fluorescence in the C group cells, and cells cultured with DHEA alone showed the maximal fluorescence intensity (Fig. 4b). Moreover, in the D group, the accumulation of MDA, hydroxyl radical, and Fe2+ content was increased, and was reduced after 1,25D3 treatment (Fig. 4c). Furthermore, SOD, GSH, and NO content were decreased by DHEA, while 1,25D3 treatment increased their content (Fig. 4c). These results implied that 1,25D3 relieved the damage caused by oxidative stress in KGN cells.

## Discussion

The mechanisms underlying development of PCOS have yet to be elucidated. Excessive ovarian androgen levels are the most common pathological feature of PCOS [3, 13]. Androgen excess may contribute to dysfunctional reproduction and metabolism in PCOS [6, 8]. Ferroptosis has been reported to be involved in multiple diseases. However, the regulatory role of ferroptosis in PCOS is unclear. Recently, Zhang et al. found that in rats, maternal hyperandrogenism, and insulin resistance caused the activation of ferroptosis in the gravid uterus and placenta [21]. To date, few studies have reported the role of DHEA-mediated ferroptosis in KGN cells. As mentioned above, VD has recently been shown to be a regulator of antioxidant capacity and iron metabolism; therefore, studying the relationship between VD and ferroptosis of KGN cells has important implications for the promotion of PCOS development and its treatment.

In the present study, a large amount of ROS production was observed in DHEA-treated KGN cells, and the level of MDA, hydroxyl radical, and Fe^2+^content increased accordingly, while SOD, GSH, and NO content were decreased as was the level of ACSL4, while the levels of GPX4 and SLC7A11 were reduced. These results are typical characteristics of ferroptosis, as reported previously [13, 17]. A high concentration of DHEA promoted the ferroptosis of KGN cells. In addition, the addition of 1,25D3 increased cell viability, enhanced intracellular GPX4 and SLC7A11 activity, and reduced intracellular ROS, MDA, hydroxyl radicals, and Fe^2+^content, while it increased SOD, GSH, and NO content, and thus inhibited ferroptosis of KGN cells induced by DHEA.

Ferroptosis, originally proposed by Dixon et al. in 2012 [2], is a newly discovered form of nonapoptotic regulated cell death (RCD) that depends on the accumulation of intracellular iron and is characterized by the formation of lipid peroxide. In patients with PCOS, androgen excess is associated with abnormal hepcidin levels, In our study, serum levels of hepcidin and testosterone were indeed negatively correlated [10]. Hyperandrogenism may decrease hepcidin levels, which might contribute to iron overload by favoring the intestinal absorption of iron. Wang et al. found that serum and ovarian MDA levels were increased in DHEA-treated rats, while an increase in ROS levels, in turn, caused mitochondrial dysfunction [19]. These studies indirectly suggested that hyperandrogenism is associated with ferroptosis. In the current study, we found that hyperandrogenism induced ferroptosis.

VDR activators attenuated cisplatin-induced ferroptosis in mouse kidneys by reducing lipid peroxidation and MDA production and reversing GPX4 downregulation [4]. VD treatment prevented the oxidative stress of renal tubule cells caused by high glucose levels, enhanced the activity of SOD, and reduced the production of MDA [22]. These outcomes may indirectly point to a potential relationship between 1,25D3 and ferroptosis. However, the effect of 1,25D3 on ferroptosis in PCOS has, to our knowledge, not to date been studied. Our work revealed that 1,25D3 administration conferred cytoprotection by inhibiting ferroptosis. Smith et al. [15] found that a high dose of 1,25D3 significantly reduced plasma hepcidin concentrations in healthy adults within 1 week of administration. With sufficient VD, reduced transcription of *Hamp* may have led to decreased intracellular and circulatory hepcidin concentrations and increased membrane abundance of ferroportin, increasing iron output and resulting in decreased intracellular iron ion concentrations [1], thereby reducing the negative effects of ferroptosis on cells. The above evidence underscores the role played by 1,25D3 in cell resistance to lipid peroxidation and ferroptosis in animals and, for the first time, we demonstrated that 1,25D3 reduced the DHEA-induced ferroptosis rate of KGN cells.

This was a pioneering study that has elucidated the functions of 1,25D3, DHEA, and ferroptosis in KGN cells and described the relationships among these factors. However, there are two possible limitations in our study. Notably, the involvement of ferroptosis still needs to be verified in a large number of people with PCOS, while the precise mechanism by which 1,25D3 affects hyperandrogen-induced ferroptosis also needs to be further explored.

In conclusion, this study confirmed the protective effect of 1,25D3 on DHEA-induced ferroptosis in KGN cells. The present study provides new evidence of the function of 1,25D3 in PCOS and the relationship between 1,25D3 and ferroptosis while additionally enhancing our understanding of the androgen-induced ferroptosis of granulosa cells in PCOS, which could be helpful in revealing the pathogenesis of PCOS. However, the precise mechanism by which ferroptosis affects the pathophysiologic progress of PCOS requires further investigation. Our research highlights newly discovered relationships that can be utilized for the future development of therapeutic strategies for PCOS.

## Data Availability

Not applicable.
